# Cancer-related fatigue in patients treated with mistletoe extracts: a systematic review and meta-analysis

**DOI:** 10.1007/s00520-022-06921-x

**Published:** 2022-03-03

**Authors:** Florian Pelzer, Martin Loef, David D. Martin, Stephan Baumgartner

**Affiliations:** 1 Institute of Integrative Medicine, Witten/Herdecke University, Witten, Germany; 2 Society for Cancer Research, Arlesheim, Switzerland; 3 CHS Institut, Berlin, Germany

**Keywords:** Cancer-related fatigue, Mistletoe extract, Viscum album, Quality of life, Cancer, Meta-analysis

## Abstract

**Purpose:**

Cancer-related fatigue remains one of the most prevalent and distressing symptoms experienced by cancer patients. Effective treatments for cancer-related fatigue are needed. The objective of this meta-analysis is to determine the impact of mistletoe extracts as a pharmacological treatment for the management of cancer-related fatigue.

**Methods:**

We included randomized clinical trials (RCTs) and non-randomized studies of interventions (NRSIs) in cancer patients. Inclusion criteria were cancer-related fatigue severity or prevalence as an outcome and testing of mistletoe extracts compared to control groups. We searched Medline (EuropePMC), Embase, the Cochrane Central Register of Controlled Trials, Clinicaltrials.gov, and opengrey.org through October 2020. We assessed the risk of bias using the Cochrane risk of bias tools for RCTs and NRSIs and conducted a meta-analysis.

**Results:**

We performed one meta-analysis with 12 RCTs, including 1494 participants, and one meta-analysis with seven retrospective NRSIs, including 2668 participants. Heterogeneity between the studies was high in both meta-analyses. Most studies had a high risk of bias. A random-effects model showed for RCTs a standardized mean difference of –0.48 (95% confidence interval –0.82 to –0.14; *p* = 0.006) and for NRSIs an odds ratio of 0.36 (95% confidence interval 0.20 to 0.66; *p* = 0.0008).

**Conclusion:**

Treatment with mistletoe extracts shows a moderate effect on cancer-related fatigue of similar size to physical activity. These results need to be confirmed by more placebo-controlled trials. Future trials should investigate different treatment durations and their effect on cancer-related fatigue in post-treatment cancer survivors.

Trial registration.

This meta-analysis has been registered under the PROSPERO registration number CRD42020191967 on October 7, 2020.

**Supplementary Information:**

The online version contains supplementary material available at 10.1007/s00520-022-06921-x.

## Introduction

Cancer-related fatigue (CRF) is a symptom experienced by cancer patients. The most cited definition, provided by the National Comprehensive Cancer Network in the USA, describes CRF as a distressing, persistent, subjective sense of physical, emotional, and/or cognitive tiredness or exhaustion related to cancer or cancer treatment that is not proportional to recent physical activity and that interferes with usual functioning [1]. There is, however, no universally accepted CRF definition [2]. The pathophysiology of CRF is unclear [3], no biomarker exists for diagnosis [3], and patients find it difficult to describe the symptom [4]. CRF is therefore measured with fatigue subscales in quality of life questionnaires or with scales assessing CRF only [4].

A recent systematic review of 129 studies with 71,568 patients reported a 49% prevalence of CRF with significant heterogeneity among studies [5]. The prevalence of CRF ranged from 11 to 99%. The variation of the prevalence can be attributed to the variety of scales, with differing cut-off values, and to the different probabilities of experiencing CRF depending on the cancer type and treatment stage [5]. Patients with gastrointestinal (50%), breast (49.7%), and lymphoma (43.3%) cancers reported a higher CRF prevalence than patients with gynecological (26.2%) and prostate (26.3%) cancers [5]. Patients in late cancer stages experience more CRF (60.6%) than patients with localized cancers (46.7%) [5].

Physical activity and psychosocial interventions are currently the first-line therapy to reduce CRF [6]. Among single pharmacological treatments, to date, only methylphenidate has evidence of improving CRF, according to a meta-analysis including seven trials with a total of 661 patients [7]. There is no consensus within the European Society for Medical Oncology, however, on whether to recommend methylphenidate against CRF [6]. Effective pharmacological treatments are needed, as physical activity has only a moderate effect size [8, 9] and cannot be applied in all oncologic settings, e.g., in cachectic patients [6]. Combinations of non-pharmacological and pharmacological treatments are currently also examined to increase treatment effects [10].

Mistletoe extracts from European mistletoe (*Viscum album* L.) are used to treat CRF [11], but the evidence has not been summarized yet. Mistletoe extracts are aqueous, total plant extracts from European mistletoe, manufactured and marketed as injectable drugs with indications in oncology [12]. In most investigational settings, mistletoe extracts are injected subcutaneously two to three times a week [13]. Injected mistletoe extracts interact both with cells of the innate and adaptive immune systems [14]. Most relevant for CRF, which can be related to reduced blood cell counts and increased inflammatory markers [3], mistletoe extracts can lead to both an increase in granulocytes [14], thereby reducing risks of neutropenia-related fatigue [15] and a reduction of inflammatory markers [14], although more research in both fields is required. In clinical routine, treatment with mistletoe extracts is often individualized by adjusting the concentration, the manufacturing method, and the mistletoe type to the patient response [16]. The treatment’s effectiveness in clinical routine has been assessed in 19 (NRSIs), while standardized treatments have been evaluated in randomized clinical trials (RCTs). Clinical studies have assessed the effect of mistletoe extracts on quality of life and survival, which have been reviewed in meta-analyses recently [17, 18]. One meta-analysis showed a non-significant effect of mistletoe extracts on the fatigue subscales of quality of life questionnaires [17]. No meta-analysis, however, has assessed the impact of mistletoe extracts on CRF in NRSIs and by including all types of CRF questionnaires. The present meta-analysis will therefore determine the impact of mistletoe extracts as a pharmacological treatment for CRF in RCTs and NRSIs.

## Methods

The protocol was registered on the International Prospective Register of Systematic Reviews (PROSPERO, https://www.crd.york.ac.uk/prospero; registration no. CRD42020191967) on October 7, 2020. The Preferred Reporting Items for Systematic Reviews and Meta-analyses (PRISMA) statement was followed for reporting [19].

### Search strategy

Two reviewers searched independently peer-reviewed articles and grey literature published until October 15, 2020, in the databases Medline (EuropePMC), Embase, the Cochrane Central Register of Controlled Trials, Clinicaltrials.gov, opengrey.org, and in the database of the Society for Cancer Research (Verein für Krebsforschung, VfK). The VfK database is specialized in mistletoe publications and is accessible under www.vfk.ch/informationen/literatursuche/. All search strategies, performed with no limitations on publication date or language, are published in the supplementary data (Table S1). Content experts in the field were consulted for further literature suggestions (Table S1). Literature search results were saved and transferred to Endnote (Clarivate Analytics, Philadelphia, USA) for search management.

### Selection criteria

Records were included if they reported RCTs or NRSIs, as NRSIs assess individualized mistletoe treatment regimens not assessed in RCTs. All included studies evaluated the impact of mistletoe extracts on CRF severity or prevalence in cancer patients via either patient- or clinician-reported outcomes. Patient-reported outcome measures that assessed “fatigue” and “tiredness” were included in the meta-analysis, as both terms are included in the CRF definition of the National Comprehensive Cancer Network. The control groups received placebo, active control, or only the treatment common to both arms. Studies were excluded in which the verum group received mistletoe extracts together with other interventions in addition to the control group’s treatment.

### Data extraction

Literature selection and data extraction were both performed independently by two reviewers. Reviewers discussed discrepant results until they achieved consensus. A first set of studies was included based on screening of titles and abstracts, which then underwent full-text analysis before confirmation of the inclusion. Where multiple records were reported on the same trial, only the earliest publication with the most complete reporting was included. “Near misses” excluded after full-text analysis were specifically documented together with the reason for their exclusion. Data was extracted into an Excel spreadsheet (Microsoft Corp., Redmond, USA) designed specifically for this project. Study design, the year when the study was conducted, population characteristics (country, number of participants at baseline and included in the analysis, mean age, cancer type), intervention (mistletoe extract type, application form, therapy duration, treatment common to both groups, adverse effects), comparator, outcome measures, effect size, and source of funding each received a coding. If CRF was evaluated via quality of life questionnaires, only the results of their fatigue subscale were included in the meta-analysis. If reported numeric data was not sufficient to calculate effect sizes, or if other non-numeric data was missing, the reviewers contacted the authors.

### Risk of bias assessment

Two reviewers assessed independently the risk of bias in RCTs with the Cochrane risk-of-bias tool for randomized trials (RoB 2) and the risk of bias in NRSIs with the Risk of Bias in Non-randomized Studies of-Interventions (ROBINS-I) tool [20]. All studies were assessed according to intention-to-treat-effect analysis. Reviewers discussed discrepant results until they achieved consensus.

We did not restrict the meta-analysis to studies with a low or moderate risk of bias, as most past studies with mistletoe extracts have not been blinded and therefore automatically have a high risk of bias, due to the local reaction arising at the injection site, which has not been reproduced by a placebo yet.

### Statistical analysis

All meta-analyses, subgroup analyses, and sensitivity analyses were conducted using Review Manager 5.4 (The Cochrane Collaboration, London, UK). The meta-regressions, Egger’s test, and Duval and Tweedie’s trim-and-fill procedure were performed with R 4.0.2 (R Foundation, Vienna, Austria) including the packages *dmetar* (written by Mathias Harrer, Pim Cuijpers, Toshi Furukawa, and David Ebert; https://dmetar.protectlab.org/articles/dmetar.html) and *meta* (written by Guido Schwarzer; https://cran.r-project.org/web/packages/meta/).

#### Main outcomes

Data from RCTs and from retrospective NRSIs were analyzed separately due to the high methodological heterogeneity of the study designs, thereby following the recommendations of the Cochrane Handbook for Systematic Reviews of Interventions regarding the inclusion of NRSI [20].

For RCTs, the effect sizes are presented as standardized mean differences (SMDs) with 95% confidence intervals (CIs). The SMD was calculated as the mean of CRF score changes from baseline to post-intervention between the verum and the control group divided by the pooled standard deviation using Hedges’ correction for small study samples. Ordinal scales reported in just one RCT [21] were dichotomized to calculate odds ratios (ORs) and subsequently converted to SMD following the Hasselblad and Hedges method [22, 23]. For NRSIs, in contrast, the OR was the predominant outcome parameter and was therefore selected as a summary measure; published SMDs [24] were converted to ORs accordingly [22, 23].

The imputation of missing values is described in the supplement. If there was a choice between multiple analyses [25] or multiple time points of the outcome [24–27], a summary effect size was calculated in accordance with the literature [20]: we used an integrative approach [28] and calculated the arithmetic mean of two or more outcome values. The pooled effect estimate therefore represents an average of different types of analyses or time points of the underlying studies. To address the high heterogeneity among studies, random-effect meta-analyses were selected for the primary analysis [20].

#### Sensitivity analysis

Since a number of studies displayed effect estimates based on single post-intervention outcomes [21, 29–32] whereas others did not [15, 33–35], we tested our handling of data multiplicity by re-calculating the meta-analyses and replaced summary effects by single outcome values resulting either from different types of analysis or from the last measurement under treatment, respectively [24–27].

Since the random-effect estimate can shift towards the smaller studies in case of a small-study effect [20], we repeated the analysis with a fixed-effect model and compared the pooled effect sizes to the outcomes of primary analysis.

The only prospective NRSI [36] was not included in the main analyses, to avoid increasing the heterogeneity by mixing prospective and retrospective designs [20]. The prospective NRSI was added in the sensitivity analysis of both prospective RCTs and retrospective NRSIs. Finally, sensitivity analyses were conducted to examine sources of heterogeneity for the NRSIs.

#### Subgroup analyses

We assessed the presence of heterogeneity between the studies via the Cochrane Q test and its size by the index of heterogeneity (*I*^2^) [37]. As a rough guide, ranges of *I*^2^ between 30 and 60%, 50 and 90%, and 75 and 100% indicate moderate, substantial, and considerable heterogeneity, respectively [20]. In order to investigate possible sources of heterogeneity, we conducted subgroup analyses for multiple moderators (country, cancer type, blinding status, mistletoe extract type, control type, additional treatment, measurement instrument, risk of bias, baseline fatigue score, study size and intervention duration). In addition, we used meta-regressions to examine the impact of the intervention duration and baseline fatigue level on the effect size.

#### Publication bias

We checked for a publication bias by examining funnel plots, Egger’s test [38], and Duval and Tweedie’s trim-and-fill procedure [39].

## Results

**Fig. 1:**
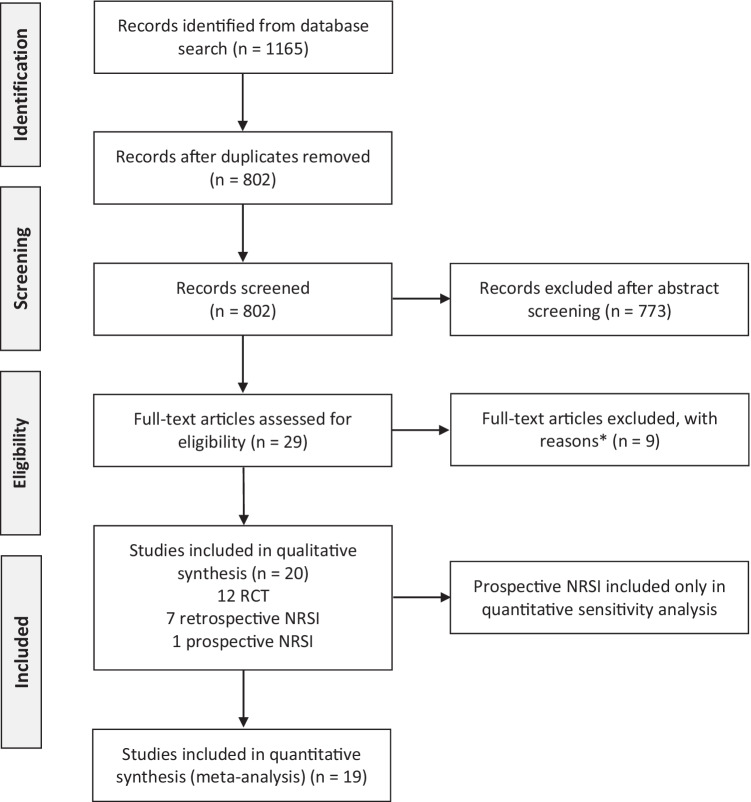
PRISMA flow diagram. The reasons for excluding records after full-text assessment (*) are shown in Table S2

We identified 802 publications by electronic searches after removing duplicates. Among the 29 full-texts analyzed, 20 studies met the inclusion criteria (Fig. 1). Reasons for the exclusion of nine “near misses” are given in the supplementary data (Table S2). We contacted nine authors and institutions/manufacturers for 12 studies, of whom five granted additional information for eight studies [25, 26, 29, 30, 33, 40–42].

### Study description

Twenty studies were included in the systematic review. There were 12 RCTs [15, 21, 25–27, 29–35], of which two were double-blinded [31, 32]. Out of eight NRSIs [24, 36, 40–45], seven had a retrospective and one a prospective design [36]. RCTs and NRSIs were analyzed in two separate meta-analyses.

#### Setting and patient characteristics

Nine studies were conducted in Germany, two in Germany and Switzerland, three in Serbia, two in Bulgaria/Russia/Ukraine, and one in China, Italy, Israel, and South Korea respectively (Table 1). CRF was measured in eleven breast, two lung, and two pancreatic cancer studies. Gastric, head and neck, and colorectal cancer as well as osteosarcoma and a group of mixed cancer types (breast, ovarian, and lung) were assessed in one study each. Mistletoe extracts were given in addition to conventional oncological treatments in all but two studies (Table 1).

**Table 1:** Characteristics of included studies (first RCTs, then NRSIs). Further information (therapy duration, baseline fatigue score, source of funding, mistletoe extract application form) is shown in Table S3

First author, publication year; country*; reference	Study design	Participants at baseline; included in fatigue-analysis; mean age		Cancer type	Intervention		Fatigue measure	Adverse effects
		Verum	Control		Verum	Control		
Bar-Sela, 2013; IL; [29]	RCT, single-center, open-label	33; 27; 63.0	39; 28; 62.0	Lung cancer (NSCLC) – inoperable IIIa, IIIb or IV	Iscador Qu; chemotherapy	Chemotherapy	EORTC QLQ-C30	5 patients; local reaction at injection site; CTCAE (V3.0) grade 1–2
Büssing, 2008; DE; [30]	RCT, multi-center, open-label	32; 32; 54.7	33; 33; 54.7	Breast cancer	Iscador M Spezial; chemo-, radiotherapy	Chemo-, radiotherapy	EORTC QLQ-C30	13/1,000 doses; chills, fever; CTCAE (V3.0) grade 1–2
Grah, 2010; DE; [25]	RCT, single-center, open-label	26; 25; 64.3	24; 24; 62.0	Lung cancer (NSCLC), IIIb or IV	Iscador Qu Spezial; chemotherapy	Chemotherapy	EORTC QLQ-C30	1.5/1,000 doses; hematoma at injection site, fever < 39 °C; heavy legs; CTCAE (V3.0) grade 1
Kim, 2012; KR; [26]	RCT, single-center, open-label	16; 15; 53.8	16; 14; 54.9	Gastric cancer Ib or II	Abnobaviscum Q; waiting for chemotherapy	Waiting for chemotherapy	EORTC QLQ-C30	25/1,000 doses; local reaction at injection site, chest pain, myalgia, dizziness, diarrhea
Longhi, 2014; IT; [35]	RCT, single-center, open-label	9; 9; 28.0	11; 11; 39.0	Osteosarcoma after second relapse	Iscador P	Etoposide	EORTC QLQ-C30	1.4/1,000 doses; local reaction at injection site, hypotension; CTCAE (V3.0) grade 1
Piao, 2004; CN; [21]	RCT, multi-center, open-label	118; 115; 52.6	115; 110; 51.7	Breast, ovarian cancer, NSCLC	Helixor A; chemotherapy	Lentinan chemotherapy	TCM	7 pats.: rubor/pruritus at injection site 4 pats.: fever 1 pat.: angioedema
Semiglasov, 2004; RU, UA, BG; [31]	RCT, multi-center, blinded	65; 65; 44.6	66; 66; 43.5	Breast cancer, pT1-3, pN0-N + , M0	Lektinol; chemotherapy	Placebo, chemotherapy	GLQ-8	12 patients with local reaction at injection site
Semiglazov, 2006; RU, UA, BG; [32]	RCT, multi-center, blinded	176; 169; 46.4	176; 168; 45.9	Breast cancer, pT1-3, pN0-N + , M0	Lektinol; chemotherapy	Placebo, chemotherapy	GLQ-8	31 patients with local reaction at injection site
Steuer-Vogt, 2006; DE; [27]	RCT, multi-center, open-label	200; 162; 55.0	199; 162; 55.0	Head and neck cancer, T1-4, N0-3, M0	Eurixor; surgery, chemo-, radiotherapy	Surgery, chemo-, radiotherapy	EORTC QLQ-C30	98 patients with rubor and prurigo; 6 patients with myalgia, insomnia, fever < 39 °C
Tröger, 2009; RS; [15]	RCT, single-center, open-label	30; 30; 48.4	31; 31; 50.8	Breast cancer, pT1-3, pN0-2, M0	Iscador M Spezial; chemotherapy	Chemotherapy	EORTC QLQ-C30	4.9/1,000 doses; local reaction at injection site
Tröger, 2014a; RS; [33]	RCT, single-center, open-label	34; 32; 50.4	31; 29; 50.8	Breast cancer, pT1-3, pN0-2, M0	Helixor A; chemotherapy	Chemotherapy	EORTC QLQ-C30	28/1,000 doses; local reactions at inj. site, allergic rhinitis; CTCAE (V3.0) grade 1–3
Tröger, 2014b; RS; [34]	RCT, single-center, open-label	110; 96; 65.0	110; 72; 61.0	Pancreatic cancer, loc. advanced or metastatic, III/IV	Iscador Qu Spezial; best support. care	Best supportive care	EORTC QLQ-C30	67 patients; local reaction at injection site
Beuth, 2008; DE; [40]	NRSI, multi-center, open-label, retrospective	227; 167; 55.1	514; 514; 54.6	Breast cancer	Helixor M, A, P; radio-, chemo-, hormone therapy	Radio-, chemo-, hormone therapy	Clinician reported	0.22/1,000 doses; local reaction at injection site, flu-like symptoms, generalized reaction
Friedel, 2009; DE, CH; [41]	NRSI, multi-center, open-label, retrospective	433; 261; 57.2	387; 145; 62.8	Colorectal cancer, primary, non-metastatic I-III	Iscador Qu, M, P, conventional therapy	Conventional therapy	Clinician reported	100 pats. with local reaction at injection site; 10 pats. with low-grade fever, dizziness, fatigue, depression, tinnitus, nausea, acute allergic reaction; CTC (V2.0) grade 1–2
Fritz, 2018; DE; [43]	NRSI, multi-center, open-label, retrospective	151; 23; 55.5	453; 77; 55.7	Breast cancer, any stage	Any mistletoe extract, radio-, chemo-, hormone ther	Radio-, chemo-, hormone therapy	EORTC QLQ-C30	not reported
Loewe-Mesch, 2008; DE; [36]	NRSI, single-center, open-label, prospective	39; 33; 47.5	43; 33; 47.5	Breast cancer, pT1-3, N0-N2; M0	Iscador M Spezial, chemotherapy	Chemotherapy only	EORTC QLQ-C30	24 patients with local reaction at injection site
Matthes, 2010; DE, CH; [42]	NRSI, multi-center, open-label, retrosepctive	201; 182; 58.2	195; 124; 63.7	Pancreatic cancer, any stage	Any Iscador, chemotherapy	Chemotherapy	Clinician reported	45 pats. with local reaction at injection site; 3 pats. with low grade fever, immune intolerance, fatigue; CTC (V2.0) grade 1–3
Oei, 2020; DE; [24]	NRSI, single-center, open-label, retrospective	129; 89; 56.4	190; 124; 60.7	Breast cancer, primary, non-metastasized	Anthroposophic mistletoe extracts, conventional therapy	Conventional therapy	EORTC QLQ-C30	not reported
Schmidt, 2007; DE; [44]	NRSI, multi-center, open-label, retrospective	710; 400; 53.0	732; 280; 57.0	Breast cancer, stage I-III	Any Iscador, radio-, chemo-, hormone ther	Radio-, chemo-, hormone therapy	Clinician reported	123 pats. with local reaction at injection site; 6 pats. with nausea, bacterial skin infection, increased neurodermatitis, fatigue, hyperactivity, diarrhea; CTC (V2.0) grade 1–2
Schumacher, 2003; DE; [45]	NRSI, multi-center, open-label, retrospective	219; 100; 60.0	470; 116; 64.0	Breast cancer, primary, non-metastasized	Eurixor, radio-, chemo-, hormone therapy	Radio-, chemo-, hormone therapy	Clinician reported	3.6/1,000 doses; local reaction at injection site, fever; CTC (V2.0) grade 1–2

^*^country abbreviations according to ISO: *IL*, Israel; *DE*, Germany; *KR*, South Korea; *IT*, Italy; *CN*, China; *RU*, Russia; *UA*, Ukraine; *BG*, Bulgaria, *RS*, Serbia; *CH*, Switzerland

#### Control conditions

The control group received placebo or active control in four trials, and only the treatment common to both arms in 16 trials (Table 1).

#### Outcome classification and measurement

CRF was measured with quality of life questionnaires in fifteen studies (Table 1). The quality of life questionnaires were the primary outcome in seven studies, the secondary outcome in six studies, while outcome classification was unspecified in two studies (Table S3). The questionnaires used were the EORTC QLQ-C30, the GLQ-8, and the Traditional Chinese Medicine (TCM) index in twelve, two, and one studies, respectively. Five NRSIs measured fatigue as an event of clinician reports (Table 1).

#### Adverse effects

The Cochrane Handbook for Systematic Reviews of Interventions defines adverse effects (AEs) as adverse events for which the causal relationship between the intervention and the event is at least a reasonable possibility [20]. AEs were reported in 18 of 20 studies (Table 1). The mean hazard rate was 1.20 ± 0.19 (standard deviation) AE per 1000 prescribed doses. Nine studies that reported AE severity had a mean hazard rate of 1.06 ± 0.25 AE grades 1–2 per 1000 prescribed doses and 0.099 ± 0.034 AE grade 3 per 1000 prescribed doses. All grade 3 AE were local reactions at the injection site. Most often reported adverse effects were local reactions at the injection site, which occurred in a mean of 24.90% ± 2.90% of participants. Systemic adverse effects included fever, flu-like symptoms, nausea, diarrhea, hypotension, and acute allergic reaction and occurred in a mean of 1.83% ± 0.16% of participants.

### Randomized controlled trials

**Fig. 2:**
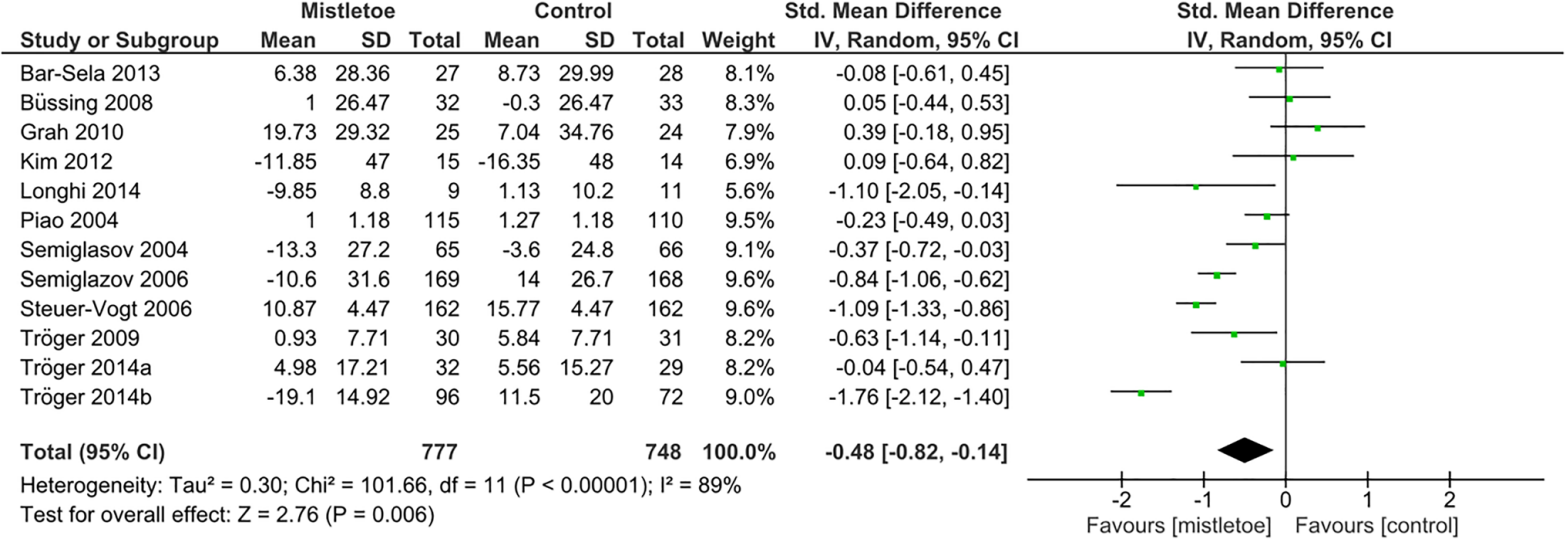
Random-effect meta-analysis pooling standardized mean differences from RCTs regarding the effect of mistletoe extracts vs. control on cancer-related fatigue

The results of the meta-analysis pooling the effect estimates from RCTs comparing mistletoe extracts and control with regard to CRF are presented in Fig. 2. Due to high heterogeneity (*I*^2^ = 89%), a random-effect meta-analysis was used to estimate a SMD of –0.48 (95% CI –0.82 to –0.14; *p* = 0.006). The subgroup analyses are displayed in Table S4: 20 of 23 pooled point estimates lie within the confidence interval of the overall SMD in Fig. 2. Fourteen of 23 SMDs were significant with *p* ≤ 0.05.

Meta-regressions on the impact of baseline fatigue and intervention duration showed tendencies corresponding to the results of the subgroup analysis but otherwise did not lead to reliable results due to the insufficient number of trials (data shown in supplement).

**Fig. 3:**
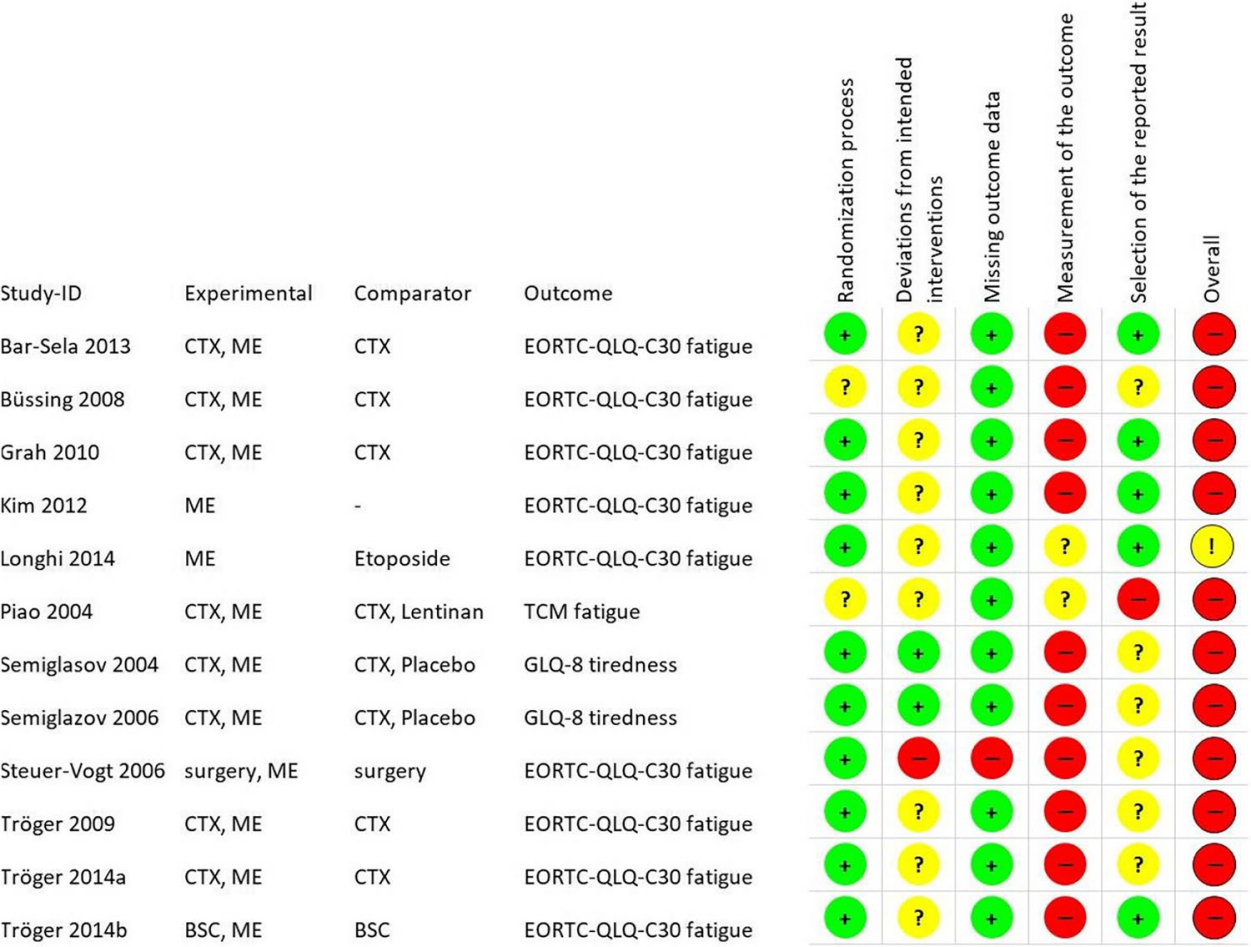
Details of risk of bias assessment of RCTs according to Cochrane RoB 2 tool (intention-to-treat). Treatments include chemotherapy (CTX), therapy with mistletoe extracts (ME), and best supportive care (BSC)

Figure 3 displays the results of the risk of bias assessment of the RCTs with the RoB 2 instrument. Of all trials, 92% had a high overall risk of bias (Fig. S3), out of which 83% are in the domain “measurement of the outcome” (Fig. S3): high risk of bias in this domain was due to the open-label measurement of CRF in eight studies [15, 25–27, 29, 30, 33, 34] and the assessment of general aches and pain within the fatigue/tiredness dimension in two studies [31, 32].

### Non-randomized studies of interventions

**Fig. 4:**
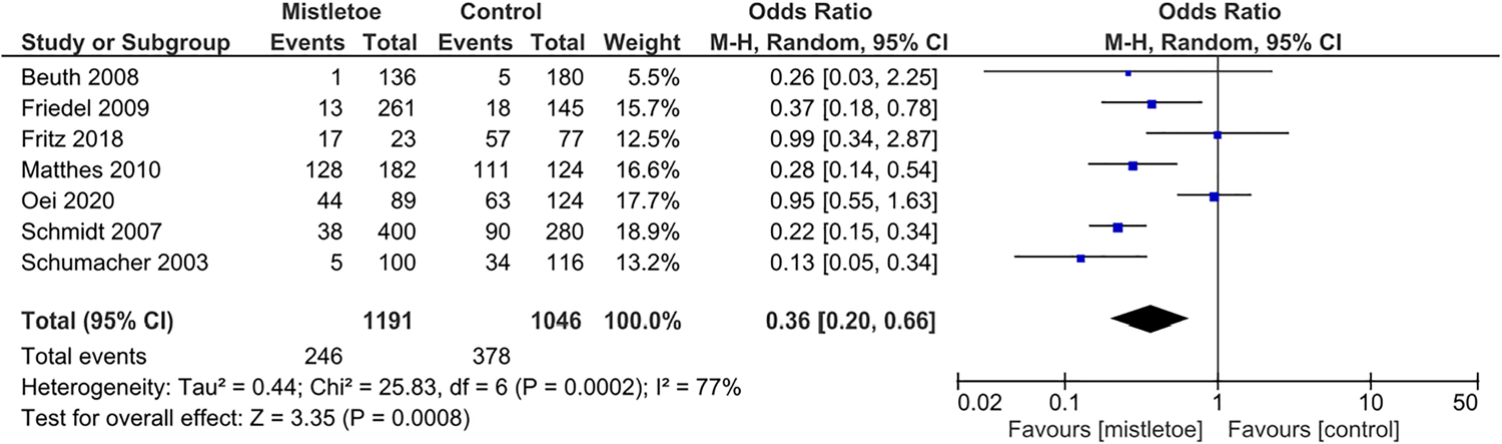
Random-effect meta-analysis pooling odds ratios from retrospective NRSIs regarding the effect of mistletoe extracts vs. control on cancer-related fatigue. OR were adjusted for baseline fatigue [24, 40, 43] or multiply adjusted

To avoid pooling prospective and retrospective designs, the prospective NRSI was included only in sensitivity analyses, shown in the supplementary data. The meta-analysis of retrospective NRSIs is displayed in Fig. 4. Due to high heterogeneity (*I*^2^ = 77%), a random-effect meta-analysis was used to estimate an OR of 0.36 (95% CI 0.20 to 0.66; *p* = 0.0008). The corresponding subgroup analyses are presented in Table S5. With the exception of NRSIs that used EORTC QLQ-C30 [24, 43], the pooled point estimates of all subgroup analyses are within the confidence interval of the overall OR in Fig. 4.

**Fig. 5:**
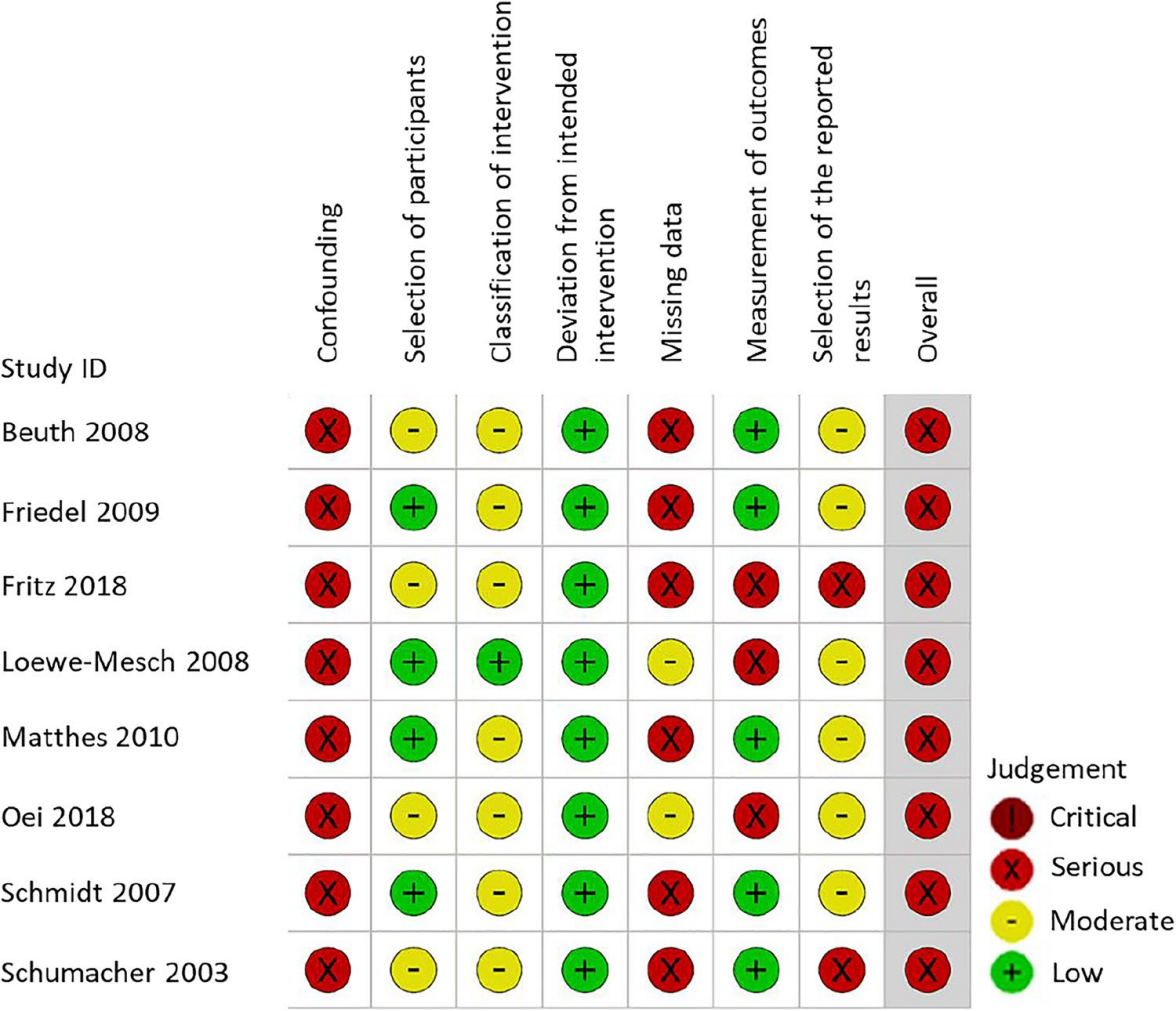
Risk of bias assessment of NRSIs according to Cochrane ROBINS-I tool (intention-to-treat)

Figure 5 shows the risk of bias assessment of NRSIs with the ROBINS-I tool. The overall risk of bias was serious for all studies. All studies were judged to be at serious risk of bias in at least two domains, but not at critical risk of bias in any domain.

### Sensitivity analysis

The overall meta-analysis regarding RCTs (Fig. 2) was repeated with alternative approaches regarding the handling of data multiplicity, study inclusion, and the analysis method (Table S6). The effect estimates proved robust against all alterations: neither the use of single post outcome values instead of mean effect estimates nor the application of a fixed-effect model resulted in a substantial impact on the effect magnitude or its significance. Most noteworthy, if the subgroup analyses were repeated with a fixed-effect model, the pooled effect sizes shifted towards the larger studies, which have a larger effect size (Table S7).

For NRSIs, multiple sensitivity analyses (Table S8) showed no relevant impact of alternative assumptions on the pooled effect estimate of the overall meta-analysis (Fig. 4).

The additional inclusion of a prospective NRSI [36] did not substantially alter the respective pooled effect sizes neither in the evaluation of all prospective studies (together with RCTs) nor in the analysis of all observational studies (together with the retrospective NRSIs) (Table S6 and S8).

### Publication bias

The meta-analysis pooling RCTs was examined for publication bias in three ways. First, the funnel plot does not resemble a symmetrical inverted funnel (Fig. S4). Second, Egger’s test displays an intercept of 3.01 (95% CI –1.16 to 7.18; *p* = 0.187). Third, Duval and Tweedie’s trim-and-fill procedure estimates no studies to be omitted on the right and three studies to be added on the left side (Fig. S5), leading to an effect estimate of SMD = –0.70 (95% CI –1.12 to –0.29, *p* = 0.0025).

For NRSIs, the studies are not symmetrically distributed in the funnel plot (Fig. S6).

## Discussion

Mistletoe extracts reduce CRF compared to control in RCTs and NRSIs. In RCT, the pooled effect estimate was SMD = –0.48, which represents a moderate effect [46]. In NRSIs, the pooled effect estimate was OR = 0.36, which in a numeric sense represents a moderate to large effect [47]. To our knowledge, the effect of mistletoe extracts on CRF has previously only been investigated as a subgroup analysis within a meta-analysis on quality of life [17]. Our results show a statistically significant but smaller effect size than the previous meta-analysis, which included 9 RCTs with 779 patients and calculated an SMD of –0.79 (95% CI –1.66 to –0.08, *p* = 0.08).

The results are robust, despite a high between-study heterogeneity. The high heterogeneity possibly derives from differences regarding the study population (e.g., cancer types, baseline fatigue levels) and methodological variations (e.g., mistletoe extract type, blinding, intervention duration, CRF measurement). Investigation of heterogeneity is difficult when there are few studies [20]. We opted to follow a pragmatic way [20] by calculating random-effect meta-analyses and testing the impact of the alternative use of fixed-effect models in the sensitivity analysis. This had no substantial influence on the SMD or the OR calculated in the overall analyses. This robustness is additionally supported by the subgroup analyses which resulted in SMDs and ORs of magnitudes that were similar to the overall pooled effect estimates.

The results have several limitations, however. Firstly, the subgroups’ explanatory power should be interpreted with caution. The number of included studies and participants often differed for subgroups of a given moderator; the uneven covariate distribution may limit the usefulness of the findings [48, 49]. In addition, there was neither clear consensus between the results of the subgroup analyses in the RCT group and two meta-regressions nor between the subgroup analyses in the RCT and the NRSI groups. This lack of consensus might be partly caused by the pooling of studies with small sample sizes [50]. For this reason, and since thresholds of the *I*^2^ statistic can be misleading for choosing a meta-analytical model [20], we tested the reliability of the subgroup SMDs by applying the fixed instead of the random-effect model. The effect estimates shifted towards the larger studies during the fixed-effect meta-analysis, indicating a small-study effect [51]. This and our subgroup analysis on sample size indicate that the effect estimates are different between small and larger trials. The small study effect should not be seen as a publication bias, however, as explained below.

Secondly, the risk of bias was high for 11 of 12 RCTs and serious for all NRSIs. In RCTs, the most prevalent high risk of bias is related to the lack of blinding. The lack of blinding is unlikely to affect the CRF measurement in RCTs, however. A recent meta-epidemiological study suggests that missing blinding has no substantial impact on treatment effect [52] and thereby cautiously questions two meta-analyses suggesting a placebo effect in CRF-treatment [53, 54]. In NRSIs, on the other hand, a recurrent serious risk of bias is observed in the domains for confounding. Numerous potential confounders for CRF have not been recorded, some because they were not known at the time of the study, e.g., physical exercise before the year 2005.

Thirdly, NRSIs can overestimate treatment effects e.g. due to selective dropout of patients experiencing only insufficient effectiveness [55]. To reduce this overestimation, we can adjust our OR by the factor for average overestimation according to Hemkens et al. [55]. This adjusted estimation would result in a moderate effect size [47] and indicate a positive, clinically relevant impact of individualized mistletoe extract treatment schemes on CRF.

Fourthly, publication bias can neither be confirmed nor excluded due to the low number of identified RCTs and to their heterogeneity. Egger’s test and Duval and Tweedie’s trim-and-fill procedure are usually recommended for a minimum of 10 studies [56]; we included 12 trials with high heterogeneity, which is why the results should be interpreted with great care [57]. While Egger’s test indicates no publication bias, the visual funnel plot examination and Duval and Tweedie’s trim-and-fill procedure may imply a bias, yet with contradictory tendencies.

Mistletoe extracts can be recommended within cancer care to treat CRF as an alternative or add-on therapy to physical activity. Mistletoe extracts have a pooled effect estimate that is comparable to other CRF interventions such as physical activity (SMD = –0.30 (95% CI –0.25 to –0.36)) [58], Tai Chi and Qigong (SMD = –0.53 (95% CI –0.97 to –0.28)) [59], and yoga (SMD = –0.30 (95% CI –0.51 to –0.08)) [60]. Patients would therefore have no disadvantage by choosing mistletoe extracts in addition to or instead of the aforementioned therapies, especially those who can no longer perform physical activities. The inclusion of mistletoe extracts in future multimodal treatment studies should be considered. Mistletoe extracts also have advantages compared to other pharmacological treatments. Firstly, mistletoe extracts have a higher effect size than methylphenidate (SMD = –0.28 (95% CI –0.44 to –0.12)) [7]. Secondly, this systematic review has found few adverse effects reported within the study durations. All reported adverse effects, such as local reactions at the injection site, are well manageable by an experienced practitioner, who should accompany treatment with mistletoe extracts. Previously published observational studies [61–63], reviews [64], and RCTs [65] identified the same types and similar hazard rates of adverse effects. Mistletoe extracts also do not seem to have a negative impact on disease-free and overall survival, but a positive one, as a recent meta-analysis showed [18].

## Conclusion

Both sources of evidence, RCTs and NRSIs, lead to effect estimates that imply a significant symptom-reducing effect of mistletoe extracts against control regarding CRF. This result is relevant for healthcare providers seeking a pharmacological treatment for CRF, in oncological settings where physical activity is not possible or where complementary CRF treatment is sought.

Future RCTs assessing the effectiveness of mistletoe extracts in CRF management need to be placebo-controlled and identify CRF as the primary outcome. Suggested topics of research are the impact of the duration of mistletoe extract treatment on CRF and the impact of mistletoe extracts on CRF in post-treatment cancer survivors. Future NRSIs need to record confounders to achieve higher certainty of evidence. NRSIs remain important sources of evidence to assess the effectiveness of individualized treatments with mistletoe extracts.

## Supplementary Information

Below is the link to the electronic supplementary material.

Supplementary file1 (DOCX 1.50 MB)

## Data Availability

The authors state that they have full control of all primary data and agree to allow the journal to review this data if requested.

## Code availability

N/A.
