# Supplementary material for: Cancer-related fatigue in patients treated with mistletoe extracts: a systematic review and meta-analysis
Source: Support Care Cancer. 2022 Mar 3;30(8):6405–18. doi: 10.1007/s00520-022-06921-x (PMC9213316; doi:10.1007/s00520-022-06921-x)
Supplement: Supplementary file 1 — Supplementary file1 (DOCX 1.50 MB) [file 520_2022_6921_MOESM1_ESM.docx]

**Supplementary Data to “Cancer-related fatigue in patients treated with mistletoe extracts:** **A systematic review and meta-analysis”**

Florian Pelzer ^1,2^, Martin Loef ^3^, David D. Martin^1^, Stephan Baumgartner^1,2^

^1^ *Institute of Integrative Medicine, Witten/Herdecke University, Witten, Germany*

^2^ *Society for Cancer Research, Arlesheim, Switzerland*

^3^ *CHS Institut, Berlin, Germany*

**Corresponding author**

Florian Pelzer, M.Sc.

E-mail: [florian.pelzer@uni-wh.de](mailto:florian.pelzer@uni-wh.de)

ORCID-Nr.: 0000-0002-8169-4539

1. Supplementary details to statistical procedures

We imputed missing means and standard deviations according to Higgins et al. (2019, chapter 6.5.2.8 ff). Standard deviations of the change from baseline were estimated with the formula:

$$\mathrm{SD}_{E, change}=\sqrt{\mathrm{SD}_{E, baseline}^{2}+\mathrm{SD}_{E,final}^{2}-\left( 2\times Corr\times\mathrm{SD}_{E, baseline}\times\mathrm{SD}_{E, final} \right)}$$

The correlation coefficient (Corr) was estimated using the maximum number of studies with sufficient data and examined for consistency. The mean correlation coefficient was estimated as Corr=0.6.

For the combination of groups we imputed missing means with:

$$M_{1,2}=\frac{N_{1}M_{1}+N_{2}M_{2}}{N_{1}+N_{2}}$$

Missing standard deviations for combined groups were calculated with:

$${SD}_{1,2}=\sqrt{\frac{\left( N_{1}-1 \right){SD}_{1}^{2}+\left( N_{2}-1 \right){SD}_{2}^{2}+\frac{N_{1}N_{2}}{N_{1}+N_{2}}\left( M_{1}^{2}+M_{2}^{2}-2M_{1}M_{2} \right)}{N_{1}+N_{2}-1}}$$

1. Additional calculations
   1. Meta-Regression

We conducted meta-regressions regarding the impact of the intervention duration and baseline fatigue level on the effect size. Those were neither significantly associated with baseline fatigue (ES -0.01 95% CI -0.03 to 0.06; p=0.52; 10 studies) nor with the intervention duration (ES -0.04 95% CI -0.08 to 0.002; p=0.06, 12 studies).

- 1. Effect sizes

SMD can be re-expressed as mean differences (MD) and OR can be re-expressed as relative risks (RR) according to [[1](#_ENREF_1)].

*Meta-analysis of RCT by pooling the MD instead of the SMD*: Mistletoe extracts reduced CRF in studies using questionnaires scaled from 100 to 0 (EORTC-QLQ-C30 and GLQ-8) with MD=8.5 (95% CI -14.99 to -2) (Fig. S1). This can be interpreted as clinically meaningful [[2](#_ENREF_2)]. Two studies [[3](#_ENREF_3), [4](#_ENREF_4)] which were double-blinded and applied GLQ8 for measuring CRF, had a pooled effect estimate of MD=-17.5 (95% CI -32.09 to -2.92; p=0.02) in contrast to MD=-6.28 (95% CI -13.23 to 0.68; p=0.08) for the open-label studies using the EORTC-QLQ-C30.


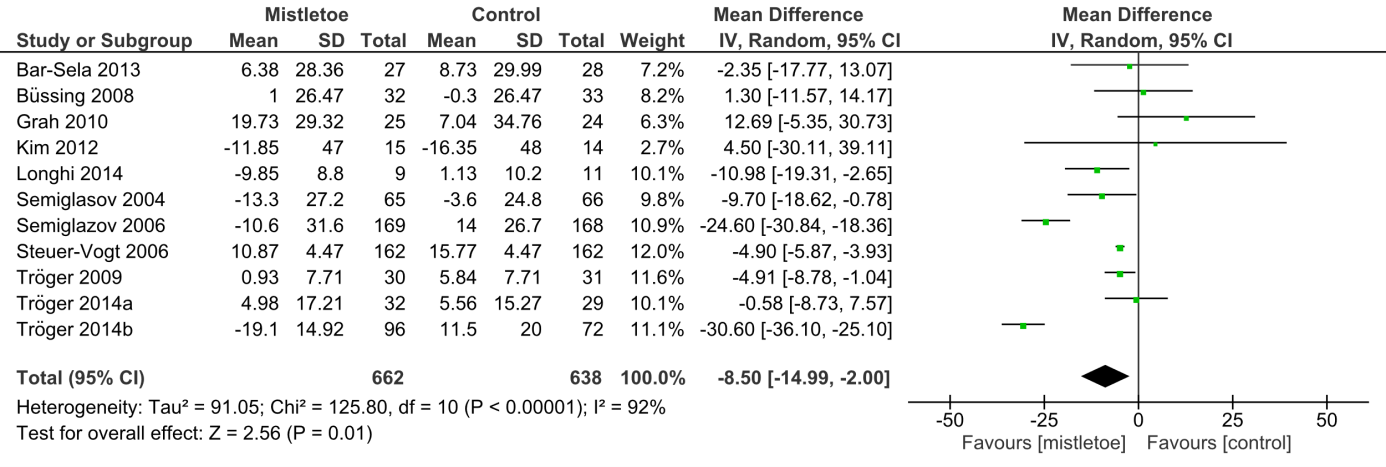


Figure S1. Random-Effect meta-analysis pooling mean differences from RCT regarding the effect of mistletoe vs. control on cancer-related fatigue

*Meta-analysis of retrospective NRSI by pooling the RR instead of the OR*: Mistletoe extracts reduced the risk of suffering from CRF by 46%, with RR=0.54 (95% CI 0.34-0.84; p=0.007) (Fig. S2). This magnitude is also comparable with a reported effect estimate of regular physical exercise on CRF [[5](#_ENREF_5)].


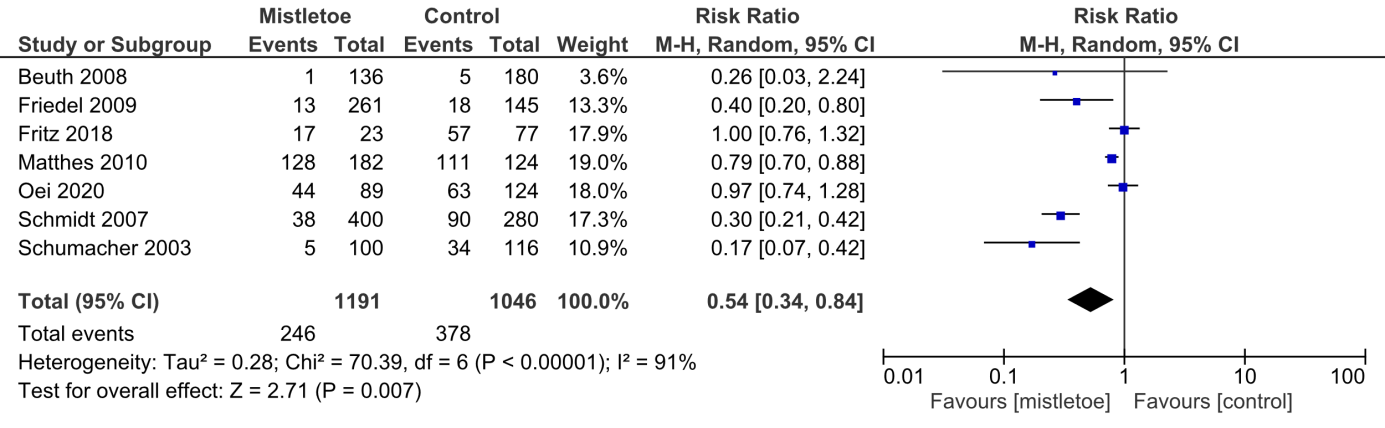


Figure S2. Random-Effect meta-analysis pooling relative risks from retrospective NRSI regarding the effect of mistletoe vs. control on cancer-related fatigue

1. Supplementary figures to risk of bias evaluation

Figure S3: Summary of risk of bias assessment of RCT in percentage according to Cochrane RoB 2 tool (intention-to-treat)

1. Supplementary figures to publication bias evaluation


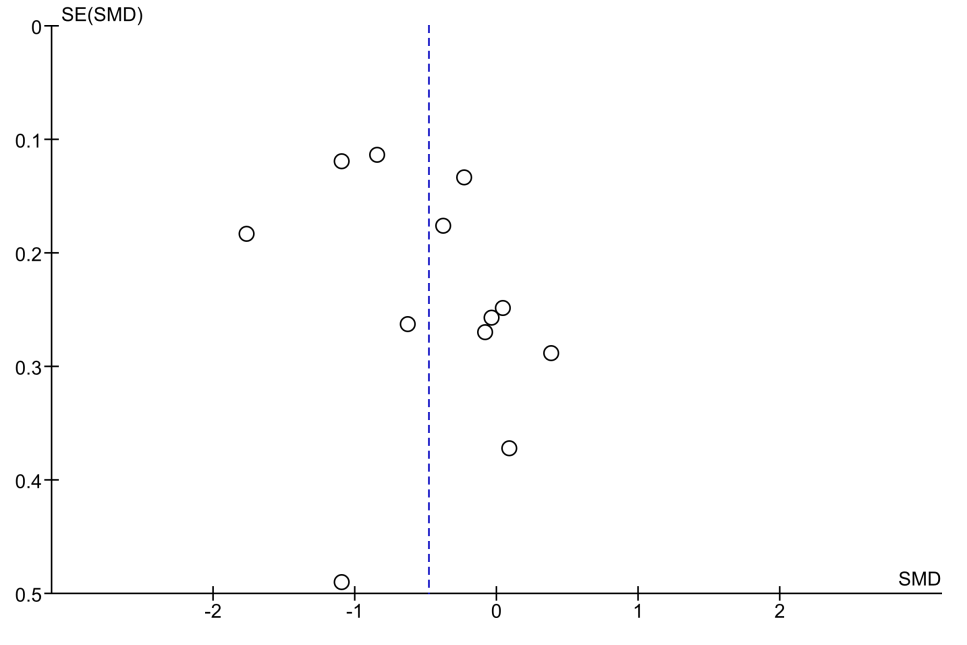


Figure S4. Funnel plot to meta-analysis in Figure 2


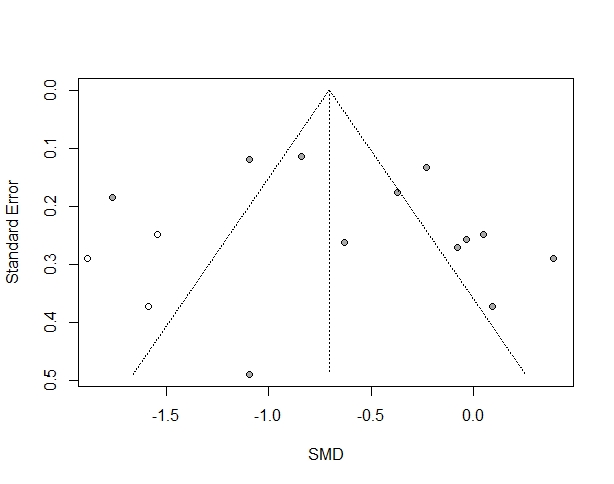


Figure S5. Visualization of the Duval & Tweedie’s trim-and-fill procedure for the RCT


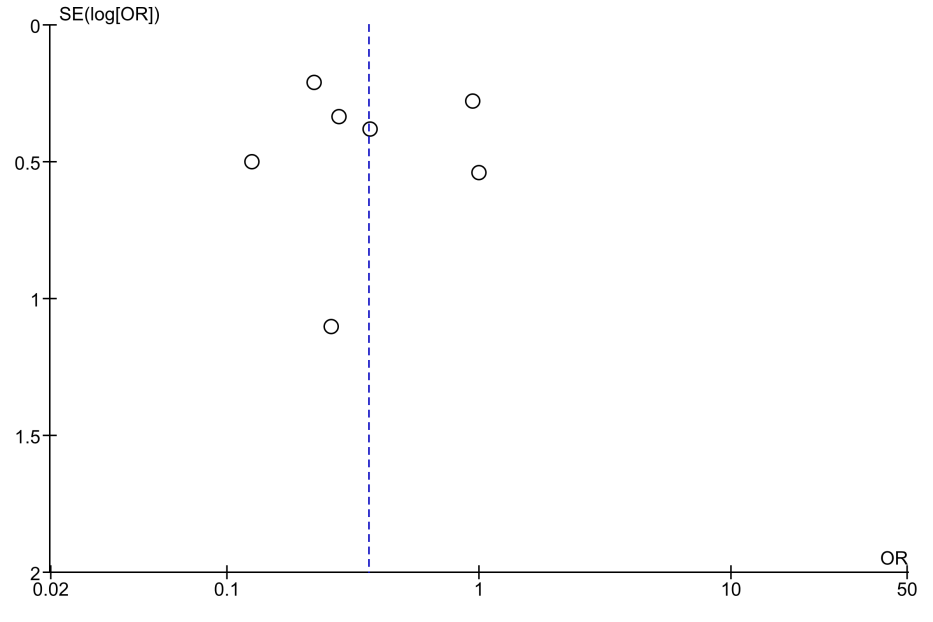


Figure S6. Funnel plot to meta-analysis in Figure 4

1. Supplementary tables

Table S1. Literature search algorithms

| **Europe PMC** (<https://europepmc.org>)  ("quality of life" OR "HRQL" OR "QOL" OR "well being" OR "Fatigue" OR "cancer related fatigue" OR "CRF" OR "asthenia" OR "müdigkeit")  AND  ("krebs" OR "cancer" OR "neoplasm" OR "tumor")  AND  ("mistletoe" OR "viscum" OR "iscador" OR "iscucin" OR "abnobaviscum" OR "helixor" OR "lektinol" OR "isorel" OR "vysorel" OR "plenosol" OR "cefalektin") |
| --- |
| **CENTRAL** (<https://www.cochranelibrary.com>)  mistletoe OR Viscum  AND  cancer OR neoplasm OR tumour  AND  quality of life OR fatigue OR QOL |
| **Embase** (<https://www.embase.com/>)  (‘quality of life’ OR ‘fatigue’ OR ‘qol’ OR ‘hrqol’ OR (‘hrql’ OR ‘patient satisfaction’ OR ‘cancer related fatigue’ OR ‘asthenia’ OR ‘müdigkeit’ OR ‘crf’)  AND  (mistletoe’ OR ‘viscum’ OR ‘mistel’ OR ‘iscador’ OR ‘abnobaviscum’ OR ‘abnoba’ OR ‘helixor’ OR ‘isorel’ OR ‘eurixor’ OR ‘iscucin’ OR ‘lektinol’)  AND  (‘cancer’ OR ‘neoplasm’ OR ‘krebs’ OR ‘tumor’ OR ‘tumour’ OR ‘carcin’) |
| **Clinicaltrials.gov** (<https://clinicaltrials.gov/>)  (mistletoe OR viscum OR Iscador OR helixor OR abnoba OR lektinol)  AND  (cancer OR neoplasm OR tumor OR tumour OR quality of life) |
| **Verein für Krebsforschung** ([www.vfk.ch/informationen/literatursuche/](http://www.vfk.ch/informationen/literatursuche/))  Sequentially searched for ‘Fatigue’, ‘Müdigkeit’, ‘Erschöpfung’ |
| **Opengrey** (<http://opengrey.org/>)  mistletoe OR viscum |
| **Content experts consulted:**  Dr. Marcus Reif (Gesellschaft für Klinische Forschung); Dr. Wilfried Tröger (Verein für Krebsforschung) |

Table S2. ‘Near-misses’ and reasons for exclusion

| Auerbach, 2005; [[6](#_ENREF_6)] | EORTC QLQ-C30 data not published, author did not respond request. |
| --- | --- |
| Bock, 2014; [[7](#_ENREF_7)] | Secondary publication of already included study |
| Büssing, 2008b; [[8](#_ENREF_8)] | HLQ-questionnaire is not validated for CRF |
| Friedel, 2012; [[9](#_ENREF_9)] | Secondary publication of already included study |
| Grah, 2019; [[10](#_ENREF_10)] | Verum group received mistletoe extracts and non-pharmacological interventions additionally to the intervention of the control group. |
| Heiny, 1997; [[11](#_ENREF_11)] | Reported questionnaire (FACT Version 3.0) has no fatigue dimension |
| Kröz, 2011; [[12](#_ENREF_12)] | No effect sizes estimable. |
| Lewicka, 2012; [[13](#_ENREF_13)] | EORTC QLQ-C30 data not published, author did not respond to request. |
| Thronicke, 2017; [[14](#_ENREF_14)] | Verum group received mistletoe extracts and non-pharmacological interventions additionally to the intervention of the control group. |

Table S3. Supplementary study characteristics

| **Reference** | | **Application form** | **Therapy duration (weeks)** | **Baseline fatigue score** | **Outcome**  **classification of the cancer-related**  **fatigue measure** | **Primary outcome (if other than cancer-related fatigue measure)** | **Source of funding** |
| --- | --- | --- | --- | --- | --- | --- | --- |
| RCT | Bar-Sela 2013 | subcutaneous | 6 | 52.1 | Primary outcome |  | Rambam Health Care Campus |
|  | Büssing 2008 | intravenous | 10 | 34.5 | Secondary outcome | Immunosuppression measure | Verein für Krebsforschung (VfK) |
|  | Grah 2010 | subcutaneous | 44 | 44.6 | Secondary outcome | Overall survival | VfK |
|  | Kim 2012 | subcutaneous | 23 | 46.6 | Primary outcome |  | Abnoba GmbH |
|  | Longhi 2014 | subcutaneous | 52 | 26.9 | Secondary outcome | Disease free survival | VfK |
|  | Piao 2004 | subcutaneous | 8 | n/a | Primary outcome |  | Helixor Heilmittel GmbH |
|  | Semiglasov 2004 | subcutaneous | 15 | 33.6 | Primary outcome |  | Madaus AG |
|  | Semiglazov 2006 | subcutaneous | 15 | n/a | Secondary outcome | Qual. of life (FACT-G) | Madaus AG |
|  | Steuer-Vogt 2006 | subcutaneous | 60 | 14 | Secondary outcome | Disease free survival | Ministry for Education and Research (Germany); biosyn GmbH |
|  | Tröger 2009 | subcutaneous | 24 | 24.2 | Primary outcome |  | VfK |
|  | Tröger 2014a | subcutaneous | 24 | 25.6 | Primary outcome |  | Helixor Heilmittel GmbH |
|  | Tröger 2014b | subcutaneous | 52 | 47.4 | Secondary outcome | Overall survival | VfK |
| NRSI | Beuth 2008 | subcutaneous | 156 | n/a | Primary outcome |  | Helixor Heilmittel GmbH |
|  | Friedel 2009 | subcutaneous | 208 | n/a | Secondary outcome | Adverse drug react. by chemo-/radiother. | VfK |
|  | Fritz 2018 | subcutaneous | n/a | n/a | Unknown |  | R. Bosch Foundation, S. Dörges Stiftung |
|  | Loewe-Mesch 2008 | subcutaneous | 11 | n/a | Unknown |  | VfK |
|  | Matthes 2010 | subcutaneous | 60 | n/a | Secondary outcome | Adverse drug react. by chemo-/radiother. | VfK |
|  | Oei 2020 | subcutaneous  intravenous | 56 | n/a | Primary outcome |  | Abnoba GmbH; Helixor Heilmittel GmbH; Iscador AG |
|  | Schmidt 2007 | subcutaneous | 208 | n/a | Secondary outcome | Adverse drug react. by chemo-/radiother. | VfK |
|  | Schumacher 2003 | subcutaneous | 39 | n/a | Primary outcome |  | biosyn GmbH |

Table S4. Subgroup analyses of the meta-analysis pooling effect estimates from RCT of mistletoe vs. control on cancer-related fatigue

| **Moderator** | **N studies** | **SMD^a^** | **95% CI** | | **Heterogeneity I^2^** | **z-score** | **p-value** |
| --- | --- | --- | --- | --- | --- | --- | --- |
| Blinding |  | | | | | | |
| Yes | 2 | –0.62 | –1.08 | –0.17 | 80% | 2.68 | 0.007 |
| No | 10 | –0.45 | –0.89 | 0 | 91% | 1.96 | 0.05 |
| Cancer type |  | | | | | | |
| Breast cancer | 5 | –0.39 | –0.76 | –0.03 | 77% | 2.13 | 0.03 |
| Other | 7 | –0.55 | –1.12 | 0.02 | 93% | 1.88 | 0.06 |
| Product type |  | | | | | | |
| Iscador | 6 | –0.52 | –1.27 | 0.24 | 92% | 1.33 | 0.18 |
| Other | 6 | –0.47 | –0.83 | –0.10 | 87% | 2.51 | 0.01 |
| Additional treatment |  | | | | | | |
| Chemotherapy | 9 | –0.23 | –0.51 | 0.05 | 76% | 1.60 | 0.11 |
| Other | 3 | –1.35 | –1.88 | –0.82 | 79% | 4.99 | < 0.00001 |
| Controls |  | | | | | | |
| Active | 4 | –0.55 | –0.93 | –0.17 | 79% | 2.86 | 0.004 |
| No Active | 8 | –0.41 | –0.95 | 0.13 | 92% | 1.48 | 0.14 |
| Country |  | | | | | | |
| Germany | 3 | –0.24 | –1.24 | 0.76 | 94% | 0.48 | 0.63 |
| Serbia | 3 | –0.82 | –1.89 | 0.25 | 94% | 1.50 | 0.13 |
| Other | 6 | –0.41 | –0.74 | –0.08 | 75% | 2.42 | 0.02 |
| Type of measurement |  | | | | | | |
| EORTC | 9 | –0.47 | –0.98 | 0.04 | 91% | 1.82 | 0.07 |
| Other | 3 | –0.49 | –0.89 | –0.08 | 85% | 2.35 | 0.02 |
| Baseline fatigue score |  | | | | | | |
| < 30 | 4 | –0.70 | –1.24 | –0.15 | 80% | 2.50 | 0.01 |
| ≥ 30 | 6 | –0.30 | –1.00 | 0.40 | 93% | 0.84 | 0.40 |
| Intervention duration |  | | | | | | |
| < 6 months | 8 | –0.30 | –0.57 | –0.03 | 73% | 2.17 | 0.03 |
| ≥ 6 months | 4 | –0.90 | –1.69 | –0.12 | 92% | 2.25 | 0.02 |
| Risk of Bias |  | | | | | | |
| ≤ 2 domains with at least some concern | 7 | –0.53 | –1.07 | 0 | 90% | 1.96 | 0.05 |
| > 2 domains with at least some concern | 5 | –0.40 | –0.89 | 0.08 | 89% | 1.62 | 0.11 |
| Study size |  | | | | | | |
| ≤ 120 patients in both arms | 7 | –0.13 | –0.44 | 0.18 | 49% | 0.82 | 0.41 |
| > 120 patients in both arms | 5 | –0.85 | –1.32 | –0.39 | 93% | 3.59 | 0.0003 |

a: random-effect meta-analysis

Table S5. Subgroup analyses of the meta-analysis pooling effect estimates from NRSI of mistletoe vs. control on cancer-related fatigue

| **Moderator** | **N studies** | **OR^a^** | **95% CI** | | **Heterogeneity I2** | **z-score** | **p-value** |
| --- | --- | --- | --- | --- | --- | --- | --- |
| Cancer type |  | | | | | | |
| Breast cancer | 5 | 0.38 | 0.16 | 0.94 | 84% | 2.10 | 0.04 |
| other | 2 | 0.31 | 0.19 | 0.51 | 0% | 4.66 | <0.00001 |
| Product type |  | | | | | | |
| Iscador | 3 | 0.26 | 0.19 | 0.35 | 0% | 8.38 | <0.00001 |
| other | 4 | 0.46 | 0.15 | 1.39 | 79% | 1.38 | 0.17 |
| Type of measurement |  | | | | | | |
| EORTC | 2 | 0.96 | 0.59 | 1.55 | 0% | 0.18 | 0.86 |
| symptom status (yes/no) | 5 | 0.24 | 0.18 | 0.32 | 0% | 9.46 | <0.00001 |
| Risk of Bias |  | | | | | | |
| ≤ 2 domains with serious concern | 5 | 0.37 | 0.19 | 0.71 | 78% | 2.98 | 0.003 |
| > 2 domains with serious concern | 2 | 0.35 | 0.05 | 2.69 | 87% | 1.01 | 0.31 |

a: random-effect meta-analysis if I^2^>0%, otherwise fixed-effect

Table S6. Sensitivity analyses of the meta-analysis in Figure 2 regarding the small-study effect (# 1), the inclusion of a prospective NRSI (# 2,4) and the handling of data multiplicity (# 3-6)

| **#** | **Moderator** | **N studies** | **SMD** | **95% CI** | | **Heterogeneity I^2^** | **z-score** | **p-value** |
| --- | --- | --- | --- | --- | --- | --- | --- | --- |
| 1 | mean post outcome [[15-17](#_ENREF_15)], fixed-effect | 12 | -0.65 | -0.76 | -0.55 | 89% | 12.21 | < 0.00001 |
| 2 | mean post outcome [[15-17](#_ENREF_15)], + [[18](#_ENREF_18)], random-effect | 13 | -0.46 | -0.78 | -0.13 | 89% | 2.76 | 0.006 |
| 3 | single time post outcome (max duration) [[15-17](#_ENREF_15)], mean of 3 analyses [[15](#_ENREF_15)], random-effect | 12 | -0.54 | -0.96 | -0.13 | 92% | 2.56 | 0.01 |
| 4 | single post outcome (max duration) [[15-17](#_ENREF_15)], + [[18](#_ENREF_18)], mean of 3 analyses [[15](#_ENREF_15)], random-effect | 13 | -0.52 | -0.91 | -0.12 | 92% | 2.56 | 0.01 |
| 5 | single post outcome (max duration) [[15-17](#_ENREF_15)], death/ drop-out as worst case analysis [[15](#_ENREF_15)], random-effect | 12 | -0.58 | -0.98 | -0.17 | 92% | 2.78 | 0.005 |
| 6 | single post outcome (max duration) [[15-17](#_ENREF_15)], MAR analysis [[15](#_ENREF_15)], random-effect | 12 | -0.48 | -0.93 | -0.03 | 93% | 2.09 | 0.04 |

Table S7. Sensitivity analyses of the RCT subgroup analyses regarding a small-study effect by applying a fixed-effect meta-analysis

| **Moderator** | **N studies** | **SMD^a^** | **95% CI** | | **Heterogeneity I^2^** | **z-score** | **p-value** |
| --- | --- | --- | --- | --- | --- | --- | --- |
| Blinding |  | | | | | | |
| Yes | 2 | –0.70 | –0.89 | –0.51 | 80% | 7.34 | < 0.00001 |
| No | 10 | –0.63 | –0.76 | –0.51 | 91% | 9.77 | < 0.00001 |
| Cancer type |  | | | | | | |
| Breast cancer | 5 | –0.55 | –0.71 | –0.39 | 77% | 6.87 | < 0.00001 |
| other | 7 | –0.74 | –0.88 | –0.60 | 93% | 10.24 | < 0.00001 |
| Product type |  | | | | | | |
| Iscador | 6 | –0.68 | –0.89 | –0.48 | 92% | 6.48 | < 0.00001 |
| other | 6 | –0.64 | –0.77 | –0.52 | 87% | 10.35 | < 0.00001 |
| Additional treatment |  | | | | | | |
| chemotherapy | 9 | –0.39 | –0.51 | –0.26 | 76% | 6.02 | < 0.00001 |
| other | 3 | –1.28 | –1.48 | –1.09 | 79% | 13.10 | < 0.00001 |
| Controls |  | | | | | | |
| Active | 4 | –0.56 | –0.71 | –0.40 | 79% | 7.23 | < 0.00001 |
| No Active | 8 | –0.75 | –0.89 | –0.60 | 92% | 10.00 | < 0.00001 |
| Country |  | | | | | | |
| Germany | 3 | –0.73 | –0.92 | –0.53 | 94% | 7.20 | < 0.00001 |
| Serbia | 3 | –1.04 | –1.30 | –0.79 | 94% | 8.03 | < 0.00001 |
| other | 6 | –0.50 | –0.64 | –0.35 | 75% | 6.85 | < 0.00001 |
| Type of measurement |  | | | | | | |
| EORTC | 9 | –0.76 | –0.90 | –0.61 | 91% | 10.22 | < 0.00001 |
| other | 3 | –0.54 | –0.69 | –0.39 | 85% | 6.96 | < 0.00001 |
| Baseline fatigue score |  | | | | | | |
| < 30 | 4 | –0.87 | –1.07 | –0.68 | 80% | 8.93 | < 0.00001 |
| ≥ 30 | 6 | –0.53 | –0.72 | –0.35 | 93% | 5.61 | < 0.00001 |
| Intervention duration |  | | | | | | |
| < 6 months | 8 | –0.43 | –0.55 | –0.30 | 73% | 6.48 | < 0.00001 |
| ≥ 6 months | 4 | –1.11 | –1.29 | –0.93 | 92% | 11.97 | < 0.00001 |
| Risk of Bias |  | | | | | | |
| ≤ 2 domains with at least some concern | 7 | –0.73 | –0.88 | –0.59 | 90% | 9.74 | < 0.00001 |
| > 2 domains with at least some concern | 5 | –0.57 | –0.72 | –0.42 | 89% | 7.51 | < 0.00001 |
| Study size |  | | | | | | |
| ≤ 120 patients in both treatment arms | 7 | –0.11 | –0.33 | 0.10 | 49% | 1.01 | 0.31 |
| > 120 patients in both treatment arms | 5 | –0.82 | –0.94 | –0.70 | 93% | 13.43 | < 0.00001 |

a: fixed-effect meta-analysis

Table S8. Sensitivity analyses of the meta-analysis in Figure 5 regarding an alternative analysis model (#1), the exclusion of one study with a high risk of bias in four domains (#2), the handling of data multiplicity (#3-4), and the inclusion of a prospective NRSI (#5)

| **#** | **Moderator** | **N studies** | **OR** | **95% CI** | | **Heterogeneity I2** | **z-score** | **p-value** |
| --- | --- | --- | --- | --- | --- | --- | --- | --- |
| 1 | fixed effect model | 7 | 0.34 | 0.27 | 0.44 | 77% | 8.51 | <0.00001 |
| 2 | - [[19](#_ENREF_19)], random effect model | 6 | 0.32 | 0.17 | 0.59 | 77% | 3.66 | 0.0003 |
| 3 | Ctx vs Ctx+mistletoe extracts, mean of 3 time points [[20](#_ENREF_20)], random effect model | 7 | 0.36 | 0.21 | 0.63 | 67% | 3.56 | 0.0004 |
| 4 | Ctx vs Ctx+mistletoe extracts, single time post outcome (max duration) [[20](#_ENREF_20)], random effect model | 7 | 0.36 | 0.20 | 0.63 | 66% | 3.56 | 0.0004 |
| 5 | + [[18](#_ENREF_18)], random effect model | 8 | 0.40 | 0.23 | 0.69 | 75% | 3.28 | 0.001 |

References (for supplementary data)

1. Andrade, C. (2015) Understanding relative risk, odds ratio, and related terms: as simple as it can get. J Clin Psychiatry 76: 857-861. <https://doi.org/10.4088/JCP.15f10150>

2. Musoro, J., et al. (2019) Minimally important differences for interpreting EORTC QLQ-C30 scores in patients with advanced breast cancer. JNCI Cancer Spectr 3: <https://doi.org/10.1093/jncics/pkz037>

3. Semiglasov, V., et al. (2004) The standardised mistletoe extract PS76A2 improves QoL in patients with breast cancer receiving adjuvant CMF chemotherapy: a randomised, placebo-controlled, double-blind, multicentre clinical trial. Anticancer Res 24: 1293-1302.

4. Semiglazov, V., et al. (2006) Quality of life is improved in breast cancer patients by Standardised Mistletoe Extract PS76A2 during chemotherapy and follow-up: a randomised, placebo-controlled, double-blind, multicentre clinical trial. Anticancer Res 26: 1519-1529.

5. Park, W., et al. (2015) Factors associated with fatigue in Korean gastric cancer survivors. Korean J Fam Med 36: <https://doi.org/10.4082/kjfm.2015.36.6.328>

6. Auerbach, L., et al. (2005) Signifikant höherer Anteil aktivierter NK-Zellen durch additive Misteltherapie bei chemotherapierten Mamma-Ca-Patientinnen in einer prospektiv-randomisierten doppelblinden Studie, In R. Scheer, et al.(ed) Fortschritte in der Misteltherapie, Aktueller Stand der Forschung und klinische Anwendung., KVC Verlag, Essen, p. 543-554

7. Bock, P., et al. (2014) Targeting inflammation in cancer-related-fatigue: a rationale for mistletoe therapy as supportive care in colorectal cancer patients. Inflamm Allergy Drug Targets 13: 105-111. <https://doi.org/10.2174/1871528113666140428103332>

8. Büssing, A., et al. (2008b) Local reactions to treatments with VISCUM ALBUM L. extracts and their association with T-lymphocyte subsets and quality of life. Anticancer Res 28: 1893-1897.

9. Friedel, W., et al. Improved quality of life and survival prolongation by long-term supportive treatment with fermented mistletoe (VISCUM ALBUM L.) extract in primary non-metastatic colorectal cancer. in *Poster European Congress Integrative Medicine 2012*. 2012.

10. Grah, C., et al. (2019) *Viscum album* L. therapy effects in the context of the ACCEPT program – Companion-Study of quality of life and therapy adherence during multimodal therapy for lung cancer. Phytomedicine 61: 1. <https://doi.org/10.1016/j.phymed.2019.09.078>

11. Heiny, B. and V. Albrecht (1997) Komplementäre Therapie mit Mistellektin-1- normiertem Extrakt; Lebensqualitätstabilisierung beim fortgeschrittenen kolorektalen Karzinom. Med Welt 48: 419-423.

12. Kröz, M., et al. (2011) Mistletoe and chemotherapy responsiveness of different scales in oncological patients undergoing chemotherapy. Phytomedicine 18: 16. <https://doi.org/10.1016/j.phymed.2011.09.039>

13. Lewicka, S., et al. (2012) Attempt to assess usefulness of the salivary cortisol determination as an objective parameter to follow effects of adjuvant and palliative therapy with VISCUM ALBUM (mistletoe preparation) in women with breast cancer Endocr Rev 33:

14. Thronicke, A., et al. Health related quality of life of non-metastasized breast cancer patients receiving multimodal integrative concepts (poster) in *Quality of Cancer Care*. 2017. Berlin, Germany.

15. Grah, C. (2010) Misteltherapie bei nichtkleinzelligem Bronchialkarzinom. Dissertation, Charité - Universitätsmedizin Berlin

16. Kim, K., et al. (2012) Quality of life, immunomodulation and safety of adjuvant mistletoe treatment in patients with gastric carcinoma - a randomized, controlled pilot study. BMC Complement Altern Med 12: <https://doi.org/10.1186/1472-6882-12-172>

17. Steuer-Vogt, M., et al. (2006) Influence of ML-1 standardized mistletoe extract on the quality of life in head and neck cancer patients. HNO 54: 277-286. <https://doi.org/10.1007/s00106-005-1318-y>

18. Loewe-Mesch, A., et al. (2008) Adjuvante simultane Mistel-/Chemotherapie bei Mammakarzinom–Einfluss auf Immunparameter, Lebensqualität und Verträglichkeit. Complement Med Res 15: 22-30. <https://doi.org/10.1159/000112860>

19. Fritz, P., et al. (2018) Is Mistletoe Treatment Beneficial in Invasive Breast Cancer? A New Approach to an Unresolved Problem. Anticancer Res 38: 1585-1593. <https://doi.org/10.21873/anticanres.12388>

20. Oei, S., et al. (2020) Impact of Oncological Therapy and Viscum album L Treatment on Cancer-Related Fatigue and Internal Coherence in Nonmetastasized Breast Cancer Patients. Integr Cancer Ther 19: <https://doi.org/10.1177/1534735420917211>
